# Protection from contamination by ^211^At, an enigmatic but promising alpha-particle-emitting radionuclide

**DOI:** 10.1186/s40658-022-00469-9

**Published:** 2022-06-06

**Authors:** Kazunobu Ohnuki, Mitsuyoshi Yoshimoto, Hiromitsu Haba, Shino Manabe, Hiroki Takashima, Masahiro Yasunaga, Yasumasa Takenaka, Hirofumi Fujii

**Affiliations:** 1 Division of Functional Imaging, Exploratory Oncology Research and Clinical Trial Center, National Cancer Center, 6-5-1 Kashiwanoha, Kashiwa, 277-8577 Japan; 2 Nishina Center for Accelerator-Based Science, RIKEN, 2-1 Hirosawa, Wako, Saitama 351-0198 Japan; 3 Pharmaceutical Department, Hoshi University, 2-4-41, Ebara, Shinagawa, Tokyo 142-8501 Japan; 4 Research Center for Pharmaceutical Development, Graduate School of Pharmaceutical Sciences and Faculty of Pharmaceutical Sciences, Tohoku University, 6-3 Aoba, Aramaki, Aoba-ku, Sendai, 980-8578 Japan; 5 Division of Developmental Therapeutics, Exploratory Oncology Research and Clinical Trial Center, National Cancer Center, 6-5-1 Kashiwanoha, Kashiwa, 277-8577 Japan; 6 RIKEN Center for Sustainable Resource Science, Bioplastic Research Team, 2-1 Hirosawa, Wako, Saitama 351-0198 Japan

**Keywords:** ^211^At, Alpha-particle-emitting radionuclide, Protection, Volatility, Plastic film, Rubber sheet

## Abstract

**Purpose:**

^211^At, a promising alpha-particle-emitting radionuclide, can easily volatilize and contaminate the environment. To safely manage this unique alpha-particle-emitting radionuclide, we investigated the permeability of four types of plastic films and two types of rubber gloves against ^211^At and identified suitable materials that prevent contamination by ^211^At.

**Methods:**

Four types of plastic films, polyethylene, polyvinylidene chloride, polyvinyl chloride, and a laminated film, and two types of rubber gloves, latex and nitrile, were examined. Small pieces of filter paper were covered with these materials, and a drop containing 100 kBq of ^211^At was placed on them. The radioactivity of the pieces of filter paper under the materials was evaluated by measuring counts using a gamma counter and obtaining autoradiograms 3.5 h later. These experiments were also performed using ^225^Ac, ^125^I, ^111^In, ^201^Tl, and ^99m^Tc.

**Results:**

^211^At solution easily penetrated polyethylene, polyvinyl chloride, and latex rubber. Similar results were obtained for ^125^I, while other radionuclides did not penetrate films or gloves. These results suggest that halogenic radionuclides under anionic conditions are likely to penetrate plastic films and rubber gloves.

**Conclusion:**

Our evaluation revealed that, when ^211^At solution is used, the protection by polyvinylidene chloride, a laminated film, or nitrile rubber would be more effective than that by polyethylene, polyvinyl chloride, or latex rubber.

**Supplementary Information:**

The online version contains supplementary material available at 10.1186/s40658-022-00469-9.

## Introduction

Protection against radiation exposure is an important issue in the field of radiology. In nuclear medicine, unsealed radionuclides are commonly used. They can easily induce contamination in surrounding areas, placing patients and medical staff at increased risk of unnecessary radiation exposure. Careful attention must be paid to avoid unnecessary contamination due to unsealed radionuclides. Anyone using unsealed radioactive materials should wear personal protective equipment (PPE) such as protective clothes, gloves, and glasses. Additionally, such materials should be handled in radiochemical fume hoods to avoid their intake [1, 2]. However, we note that the required protection measures will depend on the type of radionuclide. Currently, radionuclide therapy, in which particle-emitting radionuclides are used, is gaining popularity. In particular, radionuclide therapy using alpha-particle-emitting radionuclides has attracted considerable attention from researchers and physicians in the field of nuclear oncology. Protection from contamination by alpha-particle-emitting radionuclides is crucial because alpha particles can severely damage tissues in which they accumulate. Although alpha particles themselves can be blocked by a sheet of paper, the isolation of alpha-particle-emitting radionuclides is not always easy. We recently pointed out that a solution containing ^211^At, a promising alpha-particle-emitting radionuclide for targeted alpha therapy (TAT), can easily penetrate latex gloves, which are the most effective PPE against viruses [3]. When we work with radioactive materials, we wear PPE and wrap radioactive materials in plastic films to properly isolate them and avoid accidental internal radiation exposure. In this study, we investigated the ability of four types of plastic films and two types of gloves to protect against contamination due to ^211^At, compared with some other radionuclides, to ensure safe management of this enigmatic alpha-particle-emitting radionuclide.

## Materials and methods

The following radionuclide solutions in addition to [^211^At]NaAt solution were used to evaluate the permeability of plastic films and rubber sheets: [^225^Ac]Ac(NO_3_)_3_, [^125^I]NaI, [^111^In]InCl_3_, [^201^Tl]TlCl, and [^99m^Tc]NaTcO_4_.

^225^Ac is also a promising alpha-particle-emitting radionuclide for TAT, and this radionuclide acts as a trivalent cation in a solution. ^125^I is a halogen that acts as an anion in a solution. ^111^In is a photon emitter that acts as a trivalent cation in a solution. ^201^Tl is a photon emitter with a main energy of 70.3 keV, which is similar to that of characteristic X-rays emitted from ^211^At [4]. ^201^Tl acts as a cation in a solution. ^99m^Tc is a photon emitter that acts as an anion in the form of [^99m^Tc]TcO_4_^−^ in a solution.

The details of these radioactive solutions are shown in Additional file 1.

Four types of plastic films and two types of rubber gloves were tested. Polyethylene (30 µm), polyvinylidene chloride (11 µm), and polyvinyl chloride (8 µm) and laminated films of polypropylene, ethylene vinyl alcohol copolymer (EVAL™, Kuraray, Tokyo, Japan), and polyethylene (104 µm) were used as plastic films. The numbers in parentheses are the film thicknesses. The first three plastic films are commercially available and used for wrapping perishable food materials to maintain freshness. The laminated film was developed to pack dried fish flakes so that they are not damaged by oxygen and high humidity. As rubber sheets, pieces of latex rubber gloves (Diamond Grip PLUS, 63-754, Ansell, Brussels, Belgium) and nitrile rubber gloves (STERLING 5070, HALYARD, Mechanicsville, VA, USA) were used. These gloves are currently used as PPE against COVID-19 [5]. The thickness of each glove was more than 130 µm for latex and 70 µm for nitrile.

**Fig. 1:**
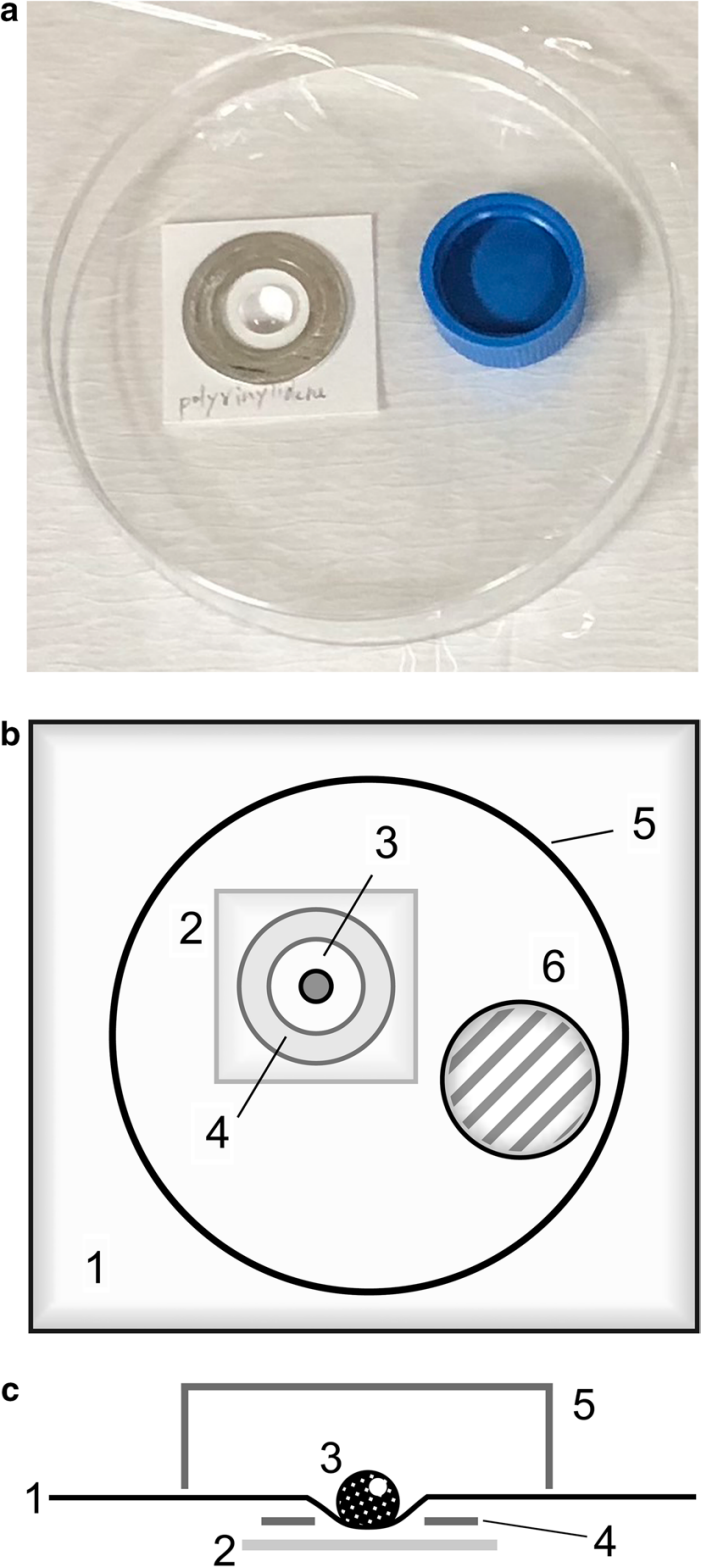
Schema of the experiments to evaluate the permeability of films and rubber. **a** Photograph indicating the configuration of a radioactive drop, sheet of film or rubber, piece of filter paper, and so on. **b** The schema of the configuration. 1: Film or rubber, 2: filtering paper, 3: radioactive drop, 4: aluminum ring (to keep the drop at the same position), 5: plastic plate (to avoid contamination by volatilized radionuclides), 6: water (to avoid vaporization of drop). **c** The schema of the section of the configuration

A three-centimeter-square piece of filter paper was covered by a sheet of plastic film or a piece of rubber cut out from a rubber glove. Fifty microliters of radionuclide solution whose radioactivity was adjusted to 100 kBq was dropped on the plastic film or rubber (Fig. 1). The plastic film or rubber was covered by a plastic Petri dish to minimize the evaporation of the radioactive solution. Each piece of filter paper under the plastic film or rubber was picked up 3.5 h later. This interval is half the half-life of ^211^At. These pieces of filter paper were placed on imaging plates (FUJIFILM, Tokyo, Japan) for 5 min and approximately 15 h. The imaging plates were scanned with an imaging plate reader (FLA-7000; FUJIFILM, Tokyo, Japan). The acquired images were analyzed using the ImageJ software (U.S. National Institutes of Health, Bethesda, MD, USA). The radioactivity of these pieces of filter paper was also measured using a gamma counter (2480 Wizard^2^; PerkinElmer, Waltham, MA, USA). These experiments were repeated three times for each radionuclide.

## Results

**Fig. 2:**
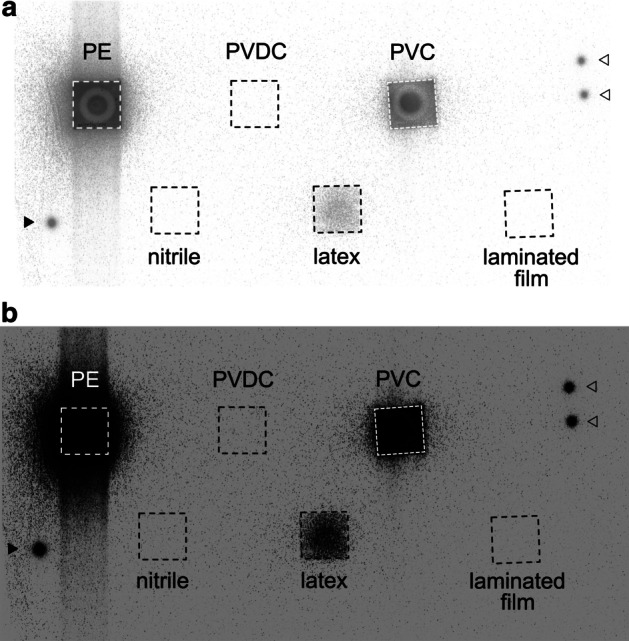
Permeability of the [^211^At]NaAt solution through films and rubber. The autoradiogram of pieces of filter paper obtained after 5-min exposure to imaging plates. PE: polyethylene, PVDC: polyvinylidene chloride, PVC: polyvinyl chloride**. a** Full-scale image, **b** overexpressed image. ^125^I drops sealed by polyvinylidene chloride film were also used as markers. The solid arrowhead indicates a ^125^I drop with 0.1 kBq and the open arrowheads indicate ^125^I drops with 0.05 kBq

When the [^211^At]NaAt solution was dropped on pieces of plastic film and sheets of rubber, strong radioactivity was detected in the filter paper under polyethylene film, polyvinyl chloride film, and latex rubber, in that order. No hot spots were detected in the pieces of filter paper under polyvinylidene chloride film, the laminated film, or the nitrile rubber (Fig. 2).

**Fig. 3:**
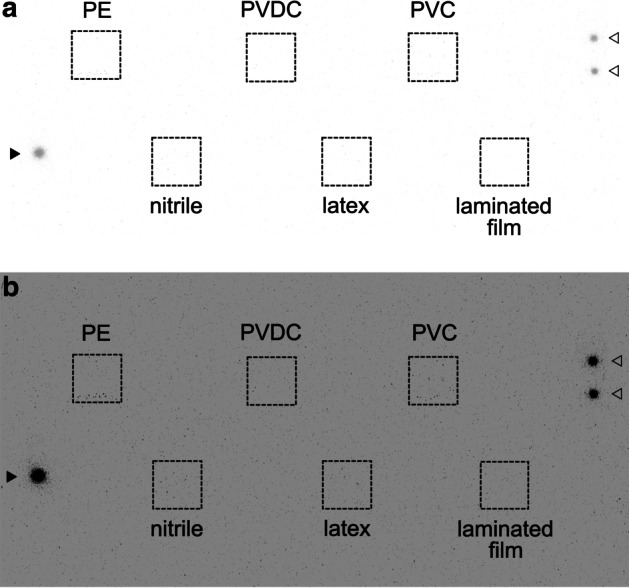
Permeability of the [^225^Ac]Ac(NO_3_)_3_ solution through films and rubber. The autoradiogram of pieces of filter paper obtained after 5-min exposure to imaging plates. **a** Full-scale image, **b** overexpressed image

When the [^225^Ac]Ac(NO_3_)_3_ solution was dropped on the materials, no significant radioactivity was detected in each piece of filter paper under films and rubber (Fig. 3, Additional file 1: Figure S1).

**Fig. 4:**
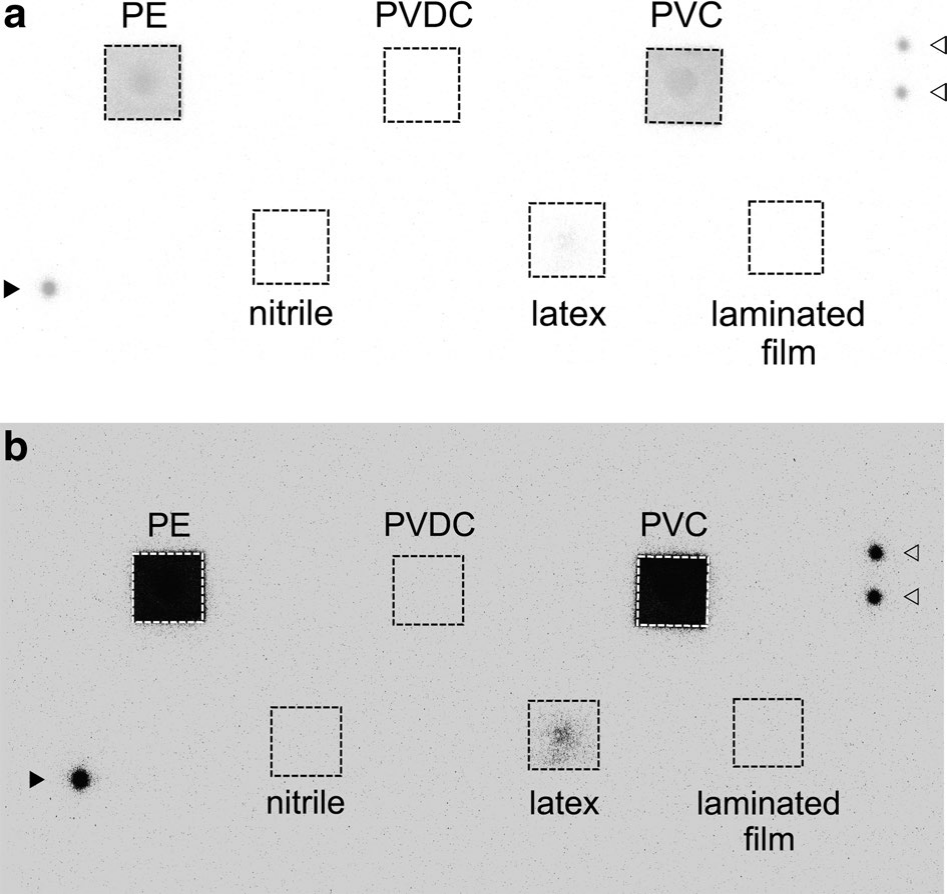
Permeability of the [^125^I]NaI solution through films and rubber. The autoradiogram obtained after 5-min exposure to imaging plates. **a** Full-scale image, **b** overexpressed image

When the [^125^I]NaI solution was dropped on the materials, strong radioactivity was detected in pieces of filter paper under polyethylene film, polyvinyl chloride film, and latex rubber, in that order. No significant radioactivity was detected in pieces of filter paper under the polyvinylidene chloride film, the laminated film, or the nitrile rubber sheet (Fig. 4). These results are similar to those of the [^211^At]NaAt solution.

When [^111^In]InCl_3_, [^201^Tl]TlCl, and [^99m^Tc]NaTcO_4_ solutions were dropped on the materials, no significant radioactivity was detected in each piece of filter paper even after 15 h of exposure (Additional file 1: Figures S2, S3 and S4).

**Fig. 5:**
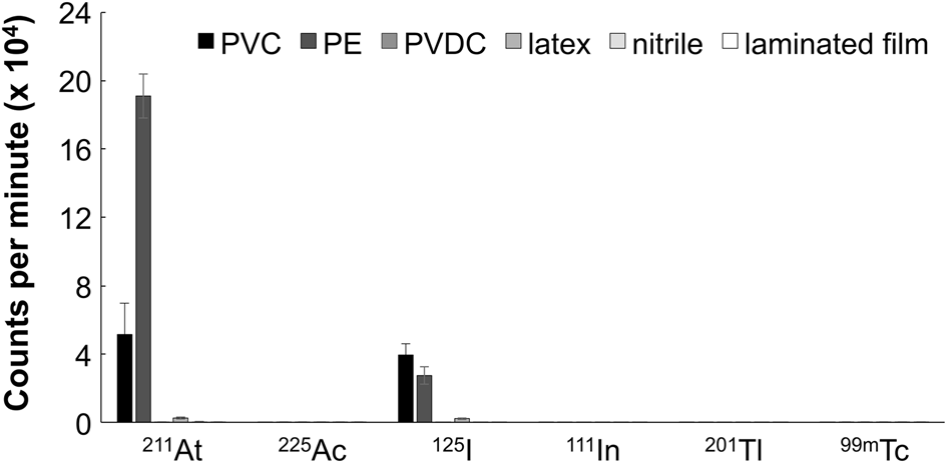
Radioactivity of pieces of filter paper counted by a gamma counter. The counts after attenuation correction are shown

The radioactivity of pieces of filter paper is shown in Fig. 5, and the originally measured data are shown in Additional file 1: Table S1.

## Discussion

^211^At, a promising alpha-particle-emitting radionuclide for TAT, is popular in Japan because of its relatively wide availability. Many researchers in Japan have been engaged in studies using radionuclides. However, this element is regarded as an enigmatic element [6], and most of its characteristics remain unclear. Previous studies have reported that ^211^At can easily volatilize and contaminate the environment [7]. Therefore, strict protection and shielding measures are essential when using this enigmatic alpha-particle-emitting radionuclide. In this study, we revealed that the permeability of the [^211^At]NaAt solution is dependent on the type of shielding material. ^225^Ac is another popular alpha-particle-emitting radionuclide, and the [^225^Ac]AcCl_3_ solution penetrated films and rubber only minimally. Because the numbers of alpha particles emitted during the single decay of ^211^At and ^225^Ac atoms are 1 and 4, respectively, ^225^Ac is a stronger alpha-particle-emitting radionuclide than ^211^At. Considering these findings, we believe that the penetration of the [^211^At]NaAt solution of films and rubber was not induced by the direct destruction of materials by emitted alpha particles. The penetration depends on the chemical properties of the shielding materials. ^225^Ac, which is cationic in a solution, and other cationic radionuclides ^111^In and ^201^Tl also failed to penetrate the shielding materials. In contrast, ^125^I, which is a halogen and is anionic in [^125^I]NaI solution in the same manner as ^211^At, showed a similar trend to that of ^211^At. However, another popular radioactive anion, [^99m^Tc]TcO_4_^−^, did not penetrate the material. These results suggest that halogenic radionuclides under anionic conditions are likely to penetrate plastic films and rubber.

Among the materials examined in this study, two types of plastic film, polyethylene and polyvinyl chloride, and the latex rubber glove were penetrated by ^211^At and were considered ineffective in protecting against the contamination due to ^211^At. However, the polyvinylidene chloride film, the unique laminated film, and the nitrile rubber glove were resistant to the penetration by the [^211^At]AtNa solution. Although the details of the differences in chemical properties of materials between these two groups, easily permeable and non-permeable for ^211^At, were unrevealed, the permeability of the [^211^At]AtNa solution was correlated with that of gas or water barrier properties according to open data [8]. The results of our additional experiment using cellophane also suggested that the gas barrier property is likely related to the mechanism of the permeability of ^211^At for plastic films and rubber gloves (data are shown in Additional file 1: Figure S5). Although further investigation is needed to determine the optimal methods to protect against the contamination by ^211^At, polyethylene is inexpensive, and bags made of this material are sold for radioactive waste disposal and are actually used in experiments with ^211^At [9]. Latex rubber gloves are also the most popular PPE and, although they fulfill special requirements for work with radioactivity, ^211^At can easily penetrate them (data are shown in Additional file 1: Figure S6 and Additional file 1: Table S2). Considering such situations, warnings should be given to people who deal with the ^211^At solution. Hence, we must wear nitrile gloves and wrap or cover specimens with polyvinylidene chloride film or a laminated film while working with compounds including ^211^At to avoid unnecessary internal radiation exposure.

## Conclusion

Our preliminary experiments indicated that the ^211^At anion can easily penetrate at least two types of plastic films, polyethylene and polyvinyl chloride, and latex rubber. The permeability of ^211^At may depend on its chemical properties as a halogen that becomes an anion in a water solution. When we deal with the ^211^At anion, we must wear nitrile gloves and wrap or cover specimens using a polyvinylidene chloride film or a laminated film.

## Supplementary Information

**Additional file 1**. Supplementary figures and tables.

## Data Availability

The data in this study are available from the corresponding author upon reasonable request.

## Abbreviations

COVID-19: Coronavirus disease 2019
PPE: Personal protective equipment
TAT: Targeted alpha therapy
PBS: Phosphate-buffered saline
