# Supplementary material for: Protection from contamination by 211At, an enigmatic but promising alpha-particle-emitting radionuclide
Source: EJNMMI Phys. 2022 Jun 6;9:39. doi: 10.1186/s40658-022-00469-9 (PMC9167904; doi:10.1186/s40658-022-00469-9)
Supplement: Supplementary file 1 — Additional file 1. Supplementary figures and tables. [file 40658_2022_469_MOESM1_ESM.pdf]

Supplementary Information

Protection from contamination by ^{211}At, an enigmatic but promising alpha-particle emitting radionuclide

Kazunobu Ohnuki[1], Mitsuyoshi Yoshimoto[1], Hiromitsu Haba[2], Shino Manabe[3, 4], Hiroki Takashima[5], Masahiro Yasunaga[5], Yasumasa Takenaka[6], Hirofumi Fujii[1]

[1]Division of Functional Imaging, Exploratory Oncology Research and Clinical Trial Center, National Cancer Center
6-5-1 Kashiwanoha, Kashiwa 277-8577 Japan
[2]Nishina Center for Accelerator-Based Science, RIKEN
2-1 Hirosawa, Wako, Saitama 351-0198 Japan
[3]Pharmaceutical Department, Hoshi University
2-4-41, Ebara, Shinagawa, Tokyo 142-8501 Japan
[4]Research Center for Pharmaceutical Development, Graduate School of Pharmaceutical Sciences and Faculty of Pharmaceutical Sciences, Tohoku University
6-3 Aoba, Aramaki, Aoba-ku, Sendai 980-8578, Japan
[5]Division of Developmental Therapeutics, Exploratory Oncology Research and Clinical Trial Center, National Cancer Center
6-5-1 Kashiwanoha, Kashiwa 277-8577 Japan
[6] Bioplastic Research Team, RIKEN Center for Sustainable Resource Science
2-1 Hirosawa, Wako, Saitama 351-0198 Japan

Correspondence to Hirofumi Fujii, M.D., Ph.D.
Division of Functional Imaging, Exploratory Oncology Research and Clinical Trial Center, National Cancer Center

6-5-1 Kashiwanoha, Kashiwa 277-8577 Japan
Phone +81-4-7134-6831, FAX +81-4-7134-6832, E-mail: hifujii@east.ncc.go.jp

Supplementary information about radioactive solutions: ^{211}At was produced via the ^{209}Bi(α, 2n)^{211}At reaction using an AVF cyclotron at the RIKEN Institute (Wako, Japan) and was freeze-dried using nitrogen gas. After transportation to the National Cancer Center, Japan, it was dissolved as [^{211}At]NaAt by phosphate-buffered saline (PBS). Its pH was 7.1 to 7.3. [^{225}Ac]Ac(NO_3)$_3$ was purchased from Global Morpho Pharma (La Chapelle-sur-Erdre, France). It was dissolved in 0.2N hydrochloric acid solution. We used this radioactive solution after dilution by PBS. As the ratio of the added PBS was small, its pH was around 2. [^{125}I]NaI was purchased from PerkinElmer (Waltham, MA, USA) and was utilized in the experiments after being diluted 1,000 times by PBS. Its pH was 7.1 to 7.3. [^{111}In]InCl_3 and [^{201}Tl]TlCl were purchased from Nihon Medi-Physics (Tokyo, Japan). [^{99m}Tc]NaTcO_4 was purchased from FUJIFILM Toyama Chemical (Tokyo, Japan). These radioactive compounds were dissolved by saline. These radioactive solutions were used in our experiments after dilution by PBS to adjust radioactivity. Their pH was also 7.1 to 7.3. Sodium cation, potassium cation, and phosphate anions were added by dilution with PBS.

Supplementary Figure 1: The permeability of the $[^{225}Ac]Ac(NO_3)_3$ solution through films and rubber. The autoradiogram obtained after 15-h exposure to imaging plates. ^{125}I drops sealed by polyvinylidene chloride film were also put as markers. The solid arrowhead indicates a ^{125}I drop with 0.1 kBq and the open arrowheads indicate ^{125}I drops with 0.05 kBq. PE: polyethylene, PVDC: polyvinylidene chloride, PVC: polyvinyl chloride.

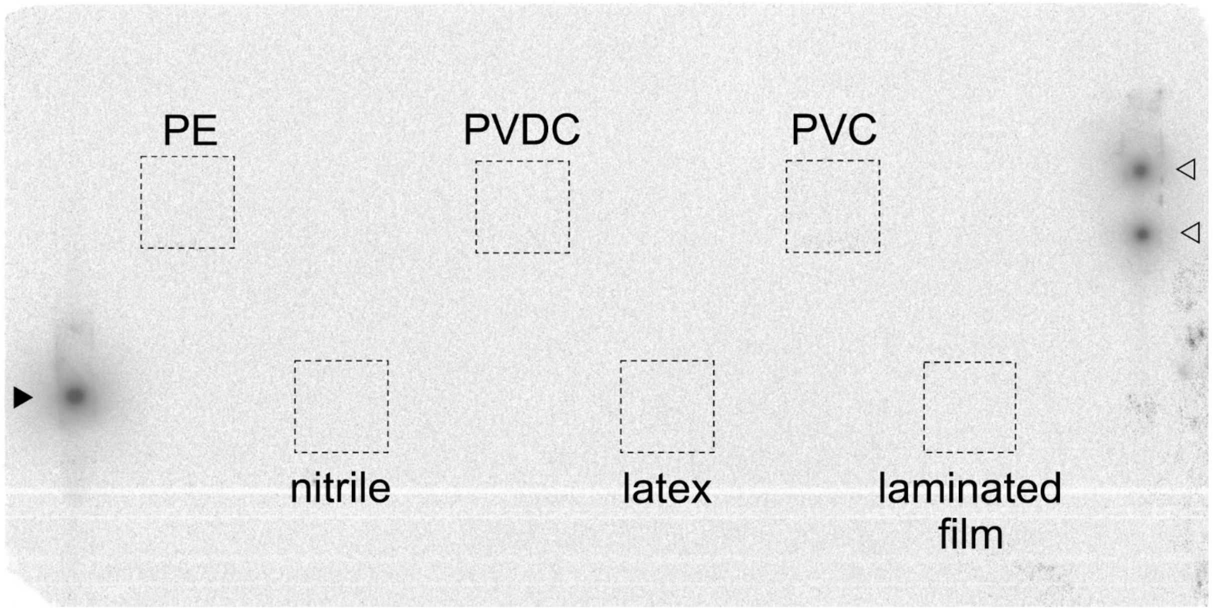

Supplementary Figure 2: The permeability of the [^{111}In]$InCl_3$ solution through films and rubber. The autoradiogram obtained after 15-h exposure to imaging plates.

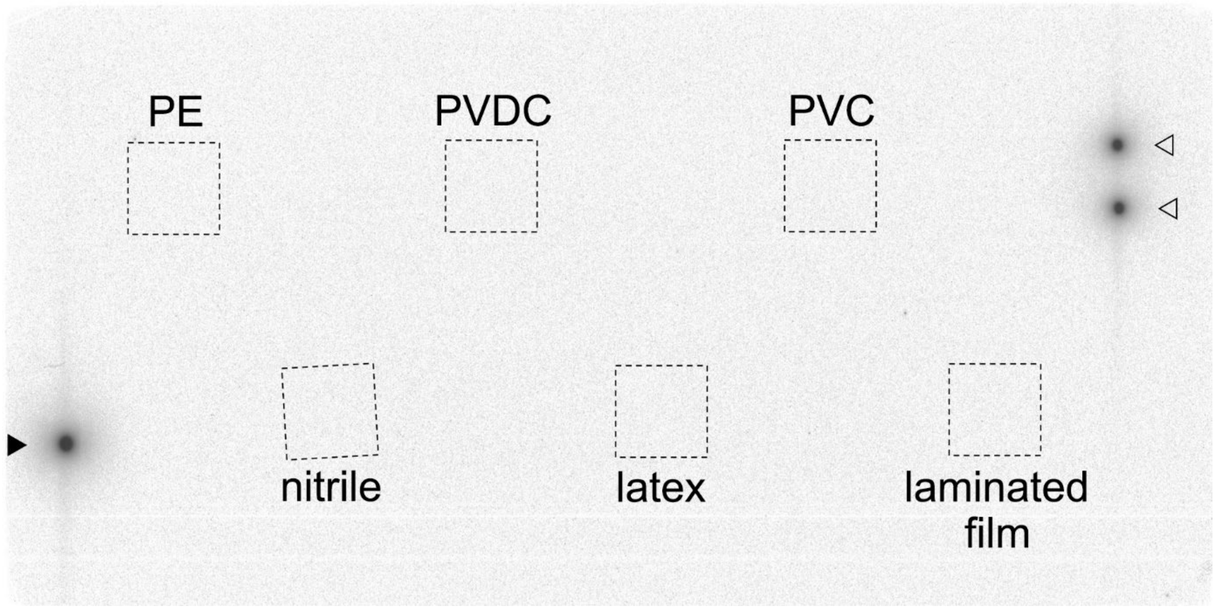

Supplementary Figure 3: The permeability of the [^{201}Tl]TlCl solution through films and rubber. The autoradiogram obtained after 15-h exposure to imaging plates.

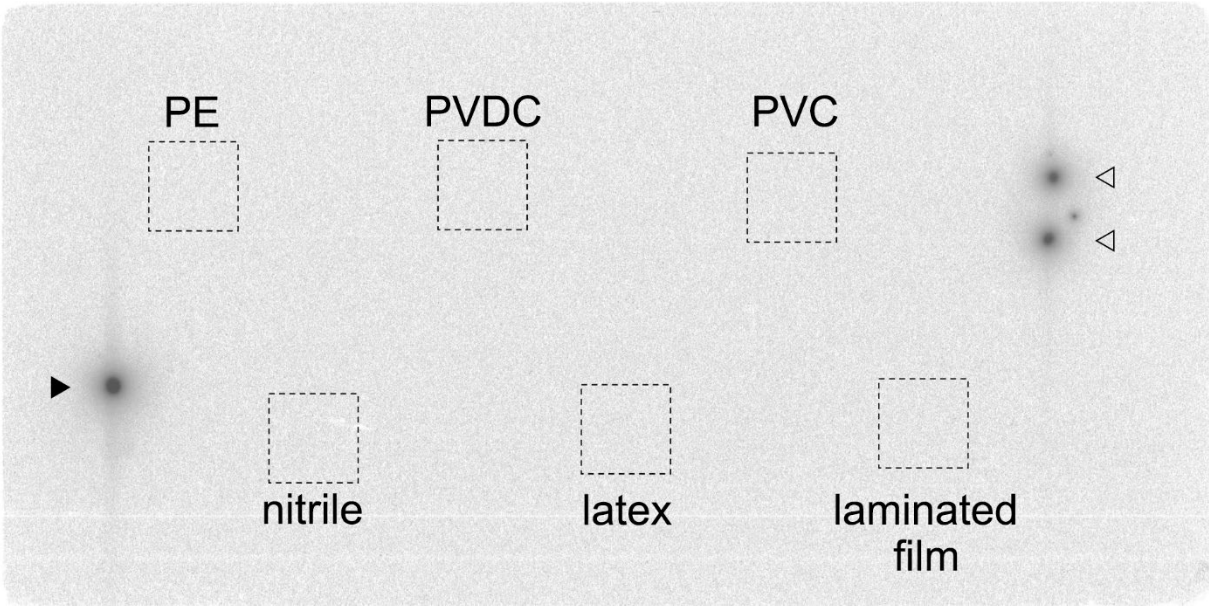

Supplementary Figure 4: The permeability of [^{99m}Tc]$NaTcO_4$ through films and rubber. The autoradiogram obtained after 15-h exposure to imaging plates.

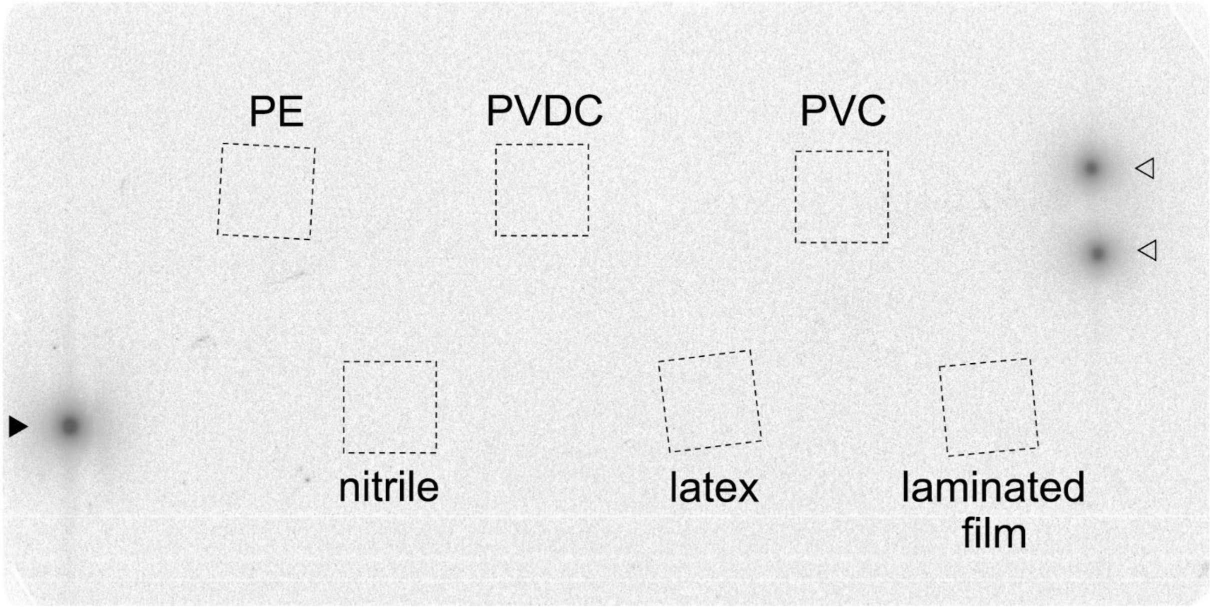

Supplementary Figure 5: The permeability of [^{211}At]NaAt through cellophane. The autoradiogram obtained after 9-h exposure to imaging plates. Although the peripheral areas of cellophane pieces show slightly increased radioactivity due to the volatilized ^{211}At, no increased activity was noted under the drop of [^{211}At]NaAt solution.

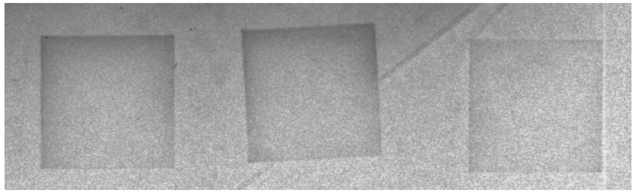

Supplementary Figure 6: The permeability of [^{211}At]NaAt through three types of latex rubber gloves. The autoradiograms obtained after 5-min and 15-h exposures to imaging plates. "Diamond Grip PLUS"(Ansell, Brussels, Belgium) and "TouchNTuff, 69-318"(Ansell) confirm to EN ISO 374-1/Type B and EN ISO 374-5 standards. "AccuTech latex rubber gloves (91-210)"(Ansell) conforms to EN421 standards in addition to EN ISO 374-1/Type B and EN ISO 374-5 standards. All of these latex rubber gloves are permeable to [^{211}At]NaAt solution.

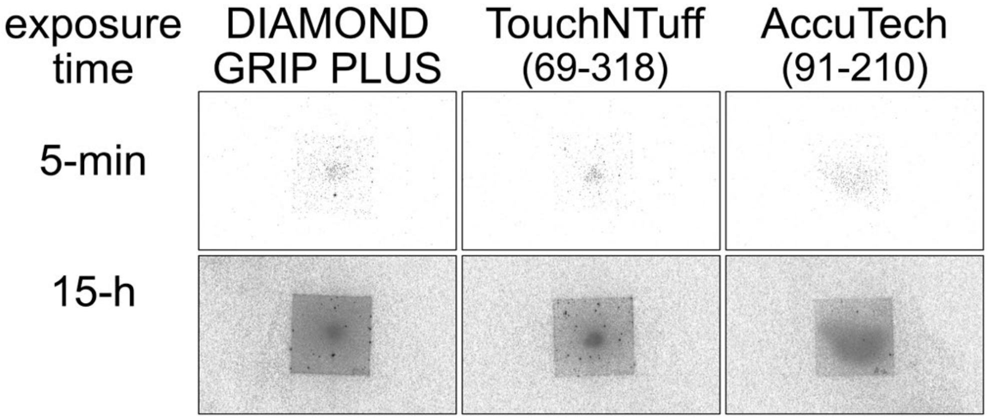

Supplementary Table 1: The radioactivity of pieces of filter paper counted by a gamma counter. The counts after attenuation correction are shown.

	[^{211}At]NaAt	[^{225}Ac]Ac(NO_3)$_3$	[^{125}I]NaI	[^{111}In]$InCl_3$	[^{201}Tl]TlCl	[^{99m}Tc]$NaTcO_4$
Polyvinyl	$(5.15 \pm 1.84) \times 10^4$	$(1.14 \pm 0.12) \times 10^2$	$(3.96 \pm 0.66) \times 10^4$	$(5.33 \pm 0.81) \times 10^1$	$(2.40 \pm 0.41) \times 10^1$	$(1.83 \pm 0.41) \times 10^2$
Polyethylene	$(1.91 \pm 0.13) \times 10^5$	$(1.17 \pm 0.12) \times 10^2$	$(2.75 \pm 0.52) \times 10^4$	$(5.23 \pm 1.04) \times 10^1$	$(2.85 \pm 0.60) \times 10^1$	$(1.58 \pm 0.40) \times 10^2$
Polyvinylidene	$(2.80 \pm 0.66) \times 10^2$	$(1.16 \pm 0.11) \times 10^2$	$(3.19 \pm 0.57) \times 10^1$	$(4.78 \pm 0.58) \times 10^1$	$(3.00 \pm 0.64) \times 10^1$	$(1.66 \pm 0.26) \times 10^2$
Latex	$(2.41 \pm 0.53) \times 10^3$	$(1.16 \pm 0.11) \times 10^2$	$(2.12 \pm 0.48) \times 10^3$	$(5.16 \pm 0.79) \times 10^1$	$(2.68 \pm 0.53) \times 10^1$	$(1.64 \pm 0.37) \times 10^2$
Nitrile	$(3.53 \pm 0.70) \times 10^2$	$(1.14 \pm 0.11) \times 10^2$	$(3.50 \pm 0.74) \times 10^1$	$(5.24 \pm 0.98) \times 10^1$	$(2.10 \pm 0.31) \times 10^1$	$(1.41 \pm 0.49) \times 10^2$
Laminated film	$(2.57 \pm 0.37) \times 10^2$	$(1.09 \pm 0.070) \times 10^2$	$(2.23 \pm 0.50) \times 10^1$	$(5.25 \pm 1.17) \times 10^1$	$(2.10 \pm 0.25) \times 10^1$	$(2.30 \pm 0.56) \times 10^2$

(counts per minute)

Supplementary Table 2: The radioactivity of pieces of filter paper under three types of latex rubber gloves counted by a gamma counter. The counts after attenuation correction are shown.

	$[^{211}At]NaAt$
DIAMOND GRIP PLUS ™ *1	(2.05 ± 0.70) x 10^3
TouchNTuff ® (69-318) *1	(1.27 ± 0.33) x 10^3
AccuTech ® (91-210) *2	(2.28 ± 0.51) x 10^3

(counts per minute)

***1**：conformable to EN ISO 374-1/Type B and EN ISO 374-5 standards

***2**：conformable to EN ISO 374-1/Type B, EN ISO 374-5, and EN421 standards
